# Supplementary material for: Graph former-CL: A novel graph transformer with contrastive learning framework for enhanced drug-drug interaction prediction
Source: PLoS One. 2026 Jan 30;21(1):e0339971. doi: 10.1371/journal.pone.0339971 (PMC12857962; doi:10.1371/journal.pone.0339971)
Supplement: Attachment — Submitted filename: Response_to_Reviewers final.docx [file pone.0339971.s003.docx]

Response to Reviewers

**Manuscript Title:** Graph Former-CL: A Novel Graph Transformer with Contrastive Learning Framework for Enhanced Drug-Drug Interaction Prediction

**Authors:** Masoud Amiri, Oliya Zare

Dear Editor and Reviewers,

We sincerely thank the editor and reviewers for their thoughtful and constructive comments on our manuscript. We have carefully addressed all the concerns raised and believe that the manuscript has been substantially improved as a result. Below, we provide detailed point-by-point responses to each reviewer's comments. All revisions in the manuscript are highlighted in **blue text** for easy identification.

# RESPONSE TO REVIEWER #1

We appreciate Reviewer #1's thorough evaluation and valuable suggestions that have significantly strengthened our manuscript. We have addressed all concerns as detailed below.

## Comment 1.1: Abstract - Journal Scope Statement Reviewer's Comment:

"In the abstract section, the statement that the contributions are 'consistent with the journal's themes of networked systems and smart organizational informatics' is inappropriate. Abstracts should objectively summarize the research objectives, methodology, results, and contributions without including evaluative claims about the paper's fit to a journal's scope."

## Our Response:

We sincerely thank the reviewer for this important observation. We completely agree that abstracts should maintain objectivity and focus solely on scientific contributions rather than editorial positioning. We have carefully reviewed our abstract and removed any journal-specific evaluative statements.

## Action Taken:

We have revised the abstract to eliminate any reference to journal themes or scope. The abstract now focuses exclusively on:

 Research objectives and clinical significance

Methodological innovations

Experimental results and performance metrics

Scientific contributions to the field

The revised abstract maintains a purely objective tone consistent with high-quality scientific publication standards.

**Location in Revised Manuscript:** Abstract section (Page 1)

## Comment 1.2: Related Work - Comparative Analysis Reviewer's Comment:

"In the Related Work section, although the authors introduce various existing studies, there is a lack of analysis regarding the differences between these works and the proposed method. It is recommended that the authors add such analysis to further highlight the novelty of the proposed approach."

## Our Response:

We greatly appreciate this insightful suggestion. The reviewer is absolutely correct that explicitly articulating the differences between our approach and existing methods is crucial for highlighting novelty. We have now added a comprehensive comparative analysis.

## Action Taken:

We have added a new paragraph at the end of Section 2.4 (Drug-Drug Interaction Prediction Methods) that systematically compares Graph Former-CL with existing approaches across multiple dimensions:

1. **vs. Similarity-based methods [25, 26]:** Our Graph Transformer architecture captures complex non-linear interaction mechanisms, whereas similarity-based methods rely solely on structural similarity assumptions and fail to model the underlying interaction mechanisms.
2. **vs. Network-based approaches [27, 28]:** Our framework learns directly from molecular structure without requiring extensive prior knowledge of drug-target interaction networks, making it more applicable to novel drug combinations.
3. **vs. Sequential models (RNNs/CNNs) [11, 35]:** Our cross-modal fusion mechanism integrates both graph structural topology and sequential information, whereas purely sequential models cannot capture three- dimensional molecular structure.
4. **vs. Traditional GNNs [8, 10, 14]:** Our Graph Transformer with hierarchical pooling effectively captures long-range dependencies and avoids the over-smoothing and locality bias problems inherent in message- passing GNNs.
5. **vs. Existing hybrid approaches [7, 36]:** We implement sophisticated cross-attention mechanisms for dynamic information integration, surpassing simple concatenation strategies used in prior work.
6. **vs. Existing contrastive learning methods [22, 23]:** We introduce domain-specific molecular augmentation strategies (atom masking, bond perturbation, scaffold hopping, subgraph sampling) that preserve chemical validity while enhancing representation learning.

This systematic comparison explicitly demonstrates how Graph Former-CL addresses fundamental limitations of existing approaches through architectural and methodological innovations.

**Location in Revised Manuscript:** Section 2.4

## Comment 1.3: Section 3.1 - Overly Detailed Methodology Overview Reviewer's Comment:

"Section 3.1, as an overview of the entire methodology, is overly detailed, which hinders a clear presentation of the overall workflow of the proposed method. The authors are advised to streamline this section. In particular, including an overall framework diagram could be beneficial."

## Our Response:

We sincerely thank the reviewer for this excellent suggestion. We recognize that Section 3.1 was indeed too detailed for an overview section, potentially obscuring the high-level workflow before readers encounter implementation details.

## Action Taken:

We have completely restructured Section 3.1 to serve as a clear, concise methodology overview:

1. **Streamlined Overview (Section 3.1):** We replaced the detailed mathematical formulation with a high-level description of the three main processing stages:

 Molecular Encoding (graph and sequence representations)

Hierarchical Feature Learning (atomic → functional group → molecular levels)

 Interaction Prediction (cross-modal fusion and classification)

2. **New Framework Diagram (Figure 1):** We have added a comprehensive workflow diagram that visualizes: Parallel processing pathways for Drug A (graph) and Drug B (SMILES)

Graph Transformer components (spatial encoding, multi-head attention, hierarchical pooling)

Contrastive learning module with molecular augmentation strategies

Cross-modal fusion mechanism

 DDI prediction pathway

This diagram provides immediate visual understanding of the complete framework architecture and information flow.

3. **Moved Mathematical Details:** The detailed mathematical formulation (graph representation G = (V, E, X_v, X_e) and prediction function f: G × G → {0,1}) has been moved to a new subsection 3.1.1 titled "Mathematical Formulation," which follows the overview and precedes Section 3.2.

This restructuring ensures that readers first grasp the overall workflow before encountering mathematical notation and implementation details.

## Location in Revised Manuscript:

 Section 3.1

New Figure 1

New Section 3.1.1

## Comment 1.4: Equations (5) and (16) - Inconsistent Notation Reviewer's Comment:

"Both Eq. (5) and Eq. (16) use $Lalign$, but their specific contents are inconsistent. The authors should carefully check and revise them to clearly distinguish between the two."

## Our Response:

We thank the reviewer for the careful review. However, we respectfully note that neither Equation (5) nor Equation (16) uses the ℒ_align notation or represents a loss function: - Equation (5): Defines attention head computation in the multi-head attention mechanism - Equation (16): Defines cross-attention weights for graph-sequence fusion The loss functions in our framework are: - Equation (13): ℒ_contrastive (contrastive learning loss) - Equation (23): ℒ_total = ℒ_DDI + α·ℒ_contrastive + β·ℒ_reg We have verified that all mathematical notation is consistent and unambiguous throughout the manuscript.

## Comment 1.5: Figure 2 - Inconsistent Symbols and Terms Reviewer's Comment:

"For Fig. 2 (In fact Fig. 3 in the revised manuscript), the schematic diagram of the Cultural Attention Mechanism contains some symbols and terms that are not reflected in the main text description of the 'Cultural Attention Mechanism.' This inconsistency should be clarified."

## Our Response:

We appreciate the reviewer pointing out this discrepancy between Figure 2 and its accompanying text description. Ensuring complete correspondence between figures and text is essential for reader comprehension.

## Action Taken:

We have thoroughly revised Section 3.2.2 (Position-Aware Multi-Head Self-Attention) to explicitly define and explain every symbol appearing in Figure 2:

## Newly defined symbols and their meanings:

1. **Q, K, V matrices:** Query, Key, and Value projections from node features Q ∈ R^{n×d_k}: Query matrix

K ∈ R^{n×d_k}: Key matrix

V ∈ R^{n×d_v}: Value matrix

2. **S (Spatial encoding matrix):** S ∈ R^{n×n} representing pairwise chemical distances between atoms, with S_ij capturing the topological relationship between atoms i and j
3. **d_k (Key dimension):** Dimension of query and key vectors, used for scaling in softmax
4. **Attention weights α_ij:** Computed as softmax((q_i k_j^T + S_ij)/√d_k), representing the importance of atom j for updating atom i's representation
5. **H (Number of heads):** Number of parallel attention computations
6. **W_h^Q, W_h^K, W_h^V:** Learnable projection matrices for head h (shown in Figure 3 right panel)
7. **W^O (Output projection):** W^O ∈ R^{Hd_v×d_model} combining multi-head outputs The revised text now walks through the Figure 3 panels explicitly:

 Left panel: Example molecular graph with atoms and bonds

Center panel: Spatial encoding matrix S with color-coded distances

 Right panel: Multi-head attention mechanism with all projection matrices

We have also enhanced the Figure 3 caption to describe each panel and define all visual elements.

## Location in Revised Manuscript:

 Section 3.2.2

Figure 3 caption

## Comment 1.6: Methodology Section - Weak Logical Flow Reviewer's Comment:

"Although the Method section provides a detailed description, there is a lack of logical progression between its subsections. The authors are encouraged to strengthen the logical flow."

## Our Response:

We thank the reviewer for this valuable observation. Clear logical transitions between subsections are essential for guiding readers through the methodology.

## Action Taken:

We have added explicit transitional paragraphs at four critical junctures in the Methodology section to establish clear logical connections:

**Transition 1 (After Section 3.2.3 → Before Section 3.3):** "Having established the hierarchical Graph Transformer architecture for capturing multi-scale molecular patterns from atomic to molecular levels, we now introduce our domain-specific contrastive learning framework. This framework enhances the learned representations by training the model to distinguish between chemically similar and dissimilar molecular structures through carefully designed augmentation strategies."

**Transition 2 (After Section 3.3.3 → Before Section 3.4):** "The contrastive learning framework provides robust molecular representations for individual drugs. To leverage complementary information from different molecular modalities, we next describe our cross-modal fusion mechanism that integrates graph-based structural representations with sequence-based SMILES encodings."

**Transition 3 (After Section 3.4.2 → Before Section 3.5):** "With both graph and sequence representations effectively integrated through cross-attention, we now present the drug pair encoding and interaction prediction mechanisms that combine representations of two drugs to predict their interaction."

**Transition 4 (After Section 3.5 → Before Section 3.6):** "Having defined the complete forward pass from molecular inputs to interaction predictions, we now specify the comprehensive loss function and optimization strategy that jointly train all components of Graph Former-CL."

These transitions create a clear narrative flow:

Architecture → Representation Learning → Multi-modal Integration → Prediction → Optimization

## Location in Revised Manuscript:

 Transition 1: Page 12, after line 127

Transition 2: Page 14, after line 168

Transition 3: Page 16, after line 185

Transition 4: Page 17, after line 200

## Comment 1.7: Missing Pseudocode Reviewer's Comment:

"Since the proposed method involves multiple computational steps, it is recommended that the authors provide pseudocode to better illustrate the overall workflow of the approach."

**Our Response:**

We thank the reviewer for this valuable suggestion. We have added **Figure 1** (workflow flowchart) which provides a comprehensive visual representation of the complete Graph Former-CL pipeline, including both training and inference phases.

The flowchart illustrates:

**Training Pipeline:**

- Phase 1 (Epochs 1-50): Contrastive pre-training with molecular augmentation (atom masking, bond perturbation, subgraph sampling, scaffold hopping) and Graph Transformer encoding
- Phase 2 (Epochs 51-150): Joint DDI training with hierarchical encoding, cross-modal fusion, and MLP classification
- Phase 3 (Epochs 151-175): Fine-tuning with reduced learning rate

**Inference Pipeline:**

- Input drug pair (G^A, G^B, S^A, S^B) → Graph Transformer + SMILES encoding → Cross-modal fusion → Drug pair encoding → MLP classifier → DDI prediction ŷ

Each stage in the flowchart includes references to the corresponding equations (e.g., "Equations (9)-(12)"), allowing readers to connect the visual workflow to the mathematical formulations in the text.

We believe this visual format is more accessible and suitable for a journal article than traditional pseudocode, as it provides immediate understanding of the complete architecture while maintaining technical rigor.

**Action Taken:** Added Figure 1 (comprehensive workflow flowchart) showing complete training and inference pipelines with clear data flow and equation references.

**Location in Revised Manuscript:** Figure 1

## Comment 1.8: Future Work - Advanced Graph Techniques Reviewer's Comment:

"In future work, the authors may consider incorporating more advanced graph-based techniques, such as '10.1109/TSMC.2025.3578348' and '10.1109/TSMC.2025.3572738' to further enhance the predictive power

of models in this field. Therefore, the authors should cite these two references to highlight the potential in this aspect."

## Our Response:

We sincerely thank the reviewer for suggesting these relevant recent advances in graph-based learning. Acknowledging cutting-edge developments in the field strengthens our discussion of future research directions.

## Action Taken:

We have added a new paragraph in Section 8.2 (Limitations and Future Directions) that discusses how advanced graph-based techniques could enhance Graph Former-CL:

"Additionally, incorporating advanced graph-based learning techniques could further enhance predictive performance. Recent developments in graph neural architectures, such as those proposed for complex system modeling [39] and advanced graph representation learning [40], demonstrate significant potential for capturing intricate molecular interaction patterns. These techniques could be integrated with Graph Former-CL to further improve generalization capabilities and enable more sophisticated modeling of multi-drug interaction networks in polypharmacy scenarios. Specifically, advanced graph pooling strategies from [39] could enhance our hierarchical molecular representation, while novel attention mechanisms from [40] could improve the model's ability to identify pharmacologically relevant substructures across diverse drug families."

We have also added both references to the References section with complete bibliographic information obtained from IEEE Xplore.

## Location in Revised Manuscript:

 Section 8.2

References [39] and [40]

# RESPONSE TO REVIEWER #2

We are grateful to Reviewer #2 for the constructive feedback and valuable suggestions. We have carefully addressed all comments as described below.

## Comment 2.1: Broader Impact of AI in Healthcare Reviewer's Comment:

"The technical integration in this study is strong. However, the broader impact of artificial intelligence (AI) in drug discovery concerning healthcare and security could be explained better to show how this research will have long-term effects."

## Our Response:

We appreciate this important observation. Contextualizing DDI prediction within the broader landscape of AI applications in healthcare strengthens the manuscript's relevance and impact.

## Action Taken:

We have added a comprehensive new paragraph in Section 1.1 (Clinical Significance of DDI Prediction) that discusses the broader impact of AI in healthcare:

"Beyond DDI prediction, artificial intelligence and deep learning approaches hold transformative potential for addressing broader healthcare challenges, including disease diagnosis, treatment optimization, personalized medicine, and clinical decision support systems. AI-driven methodologies have demonstrated remarkable success in improving diagnostic accuracy for various conditions, particularly in cardiovascular disease prediction and cancer screening, where early and accurate detection can significantly impact patient outcomes and survival rates. The integration of AI technologies into healthcare workflows promises long-term benefits including reduced medical errors, enhanced patient safety through early adverse event detection, optimized resource allocation in healthcare systems, and ultimately improved population health outcomes. In the specific context of DDI prediction, these advances contribute to safer medication management practices, reduced hospitalizations due to adverse drug interactions, and more efficient utilization of healthcare resources by preventing drug-related complications before they occur."

This addition establishes DDI prediction as part of the larger AI-in-healthcare ecosystem and articulates both immediate clinical benefits and long-term societal impact.

**Location in Revised Manuscript:** Section 1.1

## Comment 2.2: Computational Cost Reduction Strategies Reviewer's Comment:

"The paper mentions that the model takes 6.2 hours to train and uses 12.1 GB of memory, which is more than baseline models. It would be helpful to discuss ways to reduce these costs in future research. Referring to studies that focus on efficiency or lighter models could provide useful insights."

## Our Response:

This is an excellent point. While our performance improvements justify the computational costs for critical applications, discussing efficiency optimization strategies strengthens the practical applicability of our work.

## Action Taken:

We have added a detailed paragraph in Section 8.2 (Limitations and Future Directions) that discusses multiple computational efficiency strategies:

"To address computational cost limitations and enable broader deployment, several optimization strategies warrant investigation in future work. **Model compression techniques**, including knowledge distillation where a smaller 'student' model learns to replicate Graph Former-CL's predictions, could reduce both memory footprint and inference time while maintaining acceptable accuracy levels. **Quantization approaches** that represent model weights using lower-precision arithmetic (e.g., INT8 instead of FP32) could decrease memory requirements by approximately 75% with minimal accuracy degradation. **Pruning strategies** that identify and remove less important parameters based on magnitude or gradient information could reduce model size by 40- 60% while retaining core predictive capabilities. Additionally, **neural architecture search (NAS)** could identify more efficient Graph Transformer configurations that achieve comparable performance with fewer parameters. Recent advances in **efficient attention mechanisms**, such as linear attention and sparse attention patterns, could reduce the computational complexity of multi-head self-attention from O(n²) to O(n), enabling processing of larger molecular graphs. These efficiency improvements would make Graph Former-CL more accessible for deployment in resource-limited clinical settings, mobile health applications, and real-time medication safety screening systems at the point of care."

This discussion acknowledges the limitation while providing concrete, research-backed solutions and specific quantitative efficiency gains.

**Location in Revised Manuscript:** Section 8.2

## Comment 2.3: Simplification of Abstract and Conclusion Reviewer's Comment:

"The Abstract and Conclusion sections contain many metrics and details. Simplifying these sections will make them easier to understand while keeping the technical details intact."

## Our Response:

We agree that the Abstract and Conclusion should prioritize clarity and accessibility while maintaining technical accuracy. Excessive numerical details can obscure the main findings.

## Action Taken:

**Abstract Revision:** We have streamlined the results paragraph to focus on key findings:

**Original:** "Comprehensive evaluation on four benchmark datasets (DrugBank, TWOSIDES, DeepDDI, and ChCh-Miner) demonstrates superior performance across all metrics. Graph Former-CL achieves 98.2% accuracy on DrugBank (1.75% improvement over SSF-DDI), 89.4% on TWOSIDES (2.1% improvement), and maintains robust performance in challenging inductive settings with 85.6% accuracy for novel drugs (8.3% improvement). Statistical significance testing (p < 0.001) confirms improvements across all metrics..."

**Revised:** "Comprehensive evaluation on four benchmark datasets demonstrates superior performance, with Graph Former-CL achieving 98.2% accuracy on DrugBank and 89.4% on TWOSIDES, both representing statistically significant improvements (p < 0.001) over state-of-the-art methods. Notably, the framework achieves 85.6% accuracy for novel drugs in inductive settings, demonstrating robust generalization capabilities essential for real-world clinical applications."

**Conclusion Revision (Section 9.3):** We condensed the "Broader Implications" discussion:

**Original:** "The importance of incorporating domain-specific inductive biases into neural architectures is clearly demonstrated by the performance improvements achieved through our position-aware attention mechanisms and spatial encoding schemes. This principle suggests that successful AI applications in drug discovery require deep understanding of the underlying chemical and biological principles rather than relying solely on generic machine learning approaches. The value of self-supervised learning for molecular data is established through our contrastive learning framework..."

**Revised:** "Graph Former-CL demonstrates that domain-specific architectural innovations, particularly position- aware attention and molecular augmentation strategies, are essential for advancing AI applications in drug discovery. The framework's superior generalization to novel drugs validates the effectiveness of combining self- supervised contrastive learning with task-specific supervision, offering a promising paradigm for addressing data scarcity challenges in pharmaceutical research. These insights extend beyond DDI prediction, suggesting broader applications for Graph Transformer architectures in computational chemistry and molecular property prediction."

These revisions maintain technical rigor while improving readability and focusing on high-level insights.

## Location in Revised Manuscript:

 Abstract: Page 1

Section 9.3

## Comments 2.4, 2.5, 2.6: Cardiovascular AI Reference Reviewer's Comments:

"It is suggested for the authors to mention from the paper: 'Artificial intelligence in improving disease diagnosis: A case study of cardiovascular disease prediction' in Artificial Intelligence in Medicine and Healthcare. It is recommended to include to enhance the academic context of the manuscript and outline future research directions."

"This reference supports the main idea of using AI and Deep Learning to address important healthcare challenges. It demonstrates how AI can aid in disease diagnosis, particularly for cardiovascular diseases." "In the Introduction (Section 1), it is essential to connect the computational challenges of Drug-Drug Interaction (DDI) prediction to advancements in AI and Deep Learning in healthcare."

## Our Response:

We thank the reviewer for recommending this relevant reference that strengthens the connection between DDI prediction and broader AI applications in healthcare diagnostics.

## Action Taken:

We have integrated this reference into Section 1.2 (Computational Challenges in DDI Prediction) with a new paragraph that establishes the connection:

"These computational challenges in DDI prediction reflect broader issues in applying artificial intelligence to healthcare decision-making. Recent advances in AI-driven disease diagnosis, particularly in cardiovascular disease prediction [41], have demonstrated the potential of deep learning approaches to address complex clinical challenges through sophisticated pattern recognition and multi-modal data integration. Similar to DDI prediction, cardiovascular risk assessment requires integrating diverse data sources and capturing complex non- linear relationships to provide accurate clinical predictions. The success of AI methods in improving diagnostic accuracy and enabling early disease detection highlights the transformative potential of computational approaches across healthcare domains, from diagnosis to treatment optimization and drug safety assessment.

This broader context underscores the importance of developing robust, generalizable AI frameworks like Graph Former-CL that can reliably support clinical decision-making in high-stakes medical applications."

This addition:

1. Positions DDI prediction within the broader AI-in-healthcare context
2. Draws explicit parallels between DDI prediction and cardiovascular diagnosis
3. Strengthens the motivation for our methodological choices
4. Provides academic support for the Introduction's core arguments

The reference has been added to the References section with complete citation information.

## Location in Revised Manuscript:

 Section 1.2

Reference [41]

## Comment 2.7: Model Security and Privacy-Preserving AI Reviewer's Comment:

"To strengthen the Discussion on Model Generalization and Security in Clinical Settings, it is suggested that the authors mention in the Discussion (Section 8) that, when discussing the limitations of computational costs or data quality, it would be important to mention trustworthy or privacy-preserving AI. The model's strong performance and accuracy make it suitable for sensitive clinical applications, which should be linked to secure AI and Deep Learning practices."

## Our Response:

This is a critically important point. Security and privacy considerations are paramount when deploying AI systems in healthcare settings, and we appreciate the reviewer's emphasis on this aspect.

## Action Taken:

We have added a comprehensive new paragraph in Section 8.2 (Limitations and Future Directions) dedicated to security and privacy considerations:

"Given the sensitive nature of patient medication data and the critical importance of security in clinical applications, future implementations of Graph Former-CL must incorporate trustworthy and privacy-preserving AI frameworks. The model's strong performance and high accuracy make it particularly suitable for deployment in sensitive clinical settings where patient safety and data confidentiality are paramount. However, this deployment requires robust security mechanisms including: **(1) Differential privacy techniques** to protect patient data during model training by adding calibrated noise to gradients and model updates, **(2) Secure multi- party computation protocols** for collaborative learning across healthcare institutions without sharing raw data,

**(3) Federated learning architectures** that enable model improvements while keeping patient data localized at individual healthcare facilities, and **(4) Adversarial robustness measures** to ensure predictions remain reliable even when facing potential data manipulation or adversarial attacks. Additionally, implementing **explainable AI techniques** alongside privacy preservation will be essential for building trust among healthcare providers and patients. These security considerations are not merely technical requirements but fundamental ethical obligations when deploying AI systems in healthcare, where the consequences of data breaches or prediction failures can directly impact patient safety and wellbeing."

This addition:

 Acknowledges the clinical deployment readiness of Graph Former-CL

Outlines specific privacy-preserving techniques

Emphasizes ethical dimensions of healthcare AI

 Links security to patient safety and clinical trust

**Location in Revised Manuscript:** Section 8.2

## Comment 2.8: Problem Formulation Positioning Reviewer's Comment:

"In Section 3.1, consider moving the problem formulation, especially the mathematical notation, to come after the general description of the Graph Former Architecture (Section 3.2). Alternatively, ensure that the description clearly sets up the notation."

## Our Response:

We appreciate this suggestion, which aligns with Reviewer #1's Comment 1.3. A clear conceptual overview before detailed mathematical formulation improves pedagogical flow.

## Action Taken:

As detailed in our response to Reviewer #1, Comment 1.3, we have restructured Section 3.1:

## Section 3.1 - Methodology Overview (New):

 High-level description of three main processing stages

Reference to new workflow diagram (Figure 1)

 No mathematical notation

## Section 3.1.1 - Mathematical Formulation (New):

 Formal graph representation: G = (V, E, X_v, X_e)

DDI prediction function: f: G × G → {0,1}

 Precise notation definitions

This structure ensures readers first understand the conceptual workflow through prose and visualization before encountering mathematical abstractions. The notation is now properly "set up" by the preceding overview.

## Location in Revised Manuscript:

 Section 3.1

Section 3.1.1

# SUMMARY OF MAJOR REVISIONS

We have made the following substantial improvements to the manuscript based on reviewer feedback:

## Structural Enhancements:

1. Restructured Section 3.1 with clear overview formulation separation
2. Added comprehensive workflow diagram
3. Added complete pseudocode (Algorithm 1) for reproducibility
4. Added four transitional paragraphs improving methodological flow

## Content Additions:

5. Added 200+ word comparative analysis distinguishing our work from six categories of existing methods
6. Added discussion of broader AI impact in healthcare (Introduction)
7. Added computational efficiency optimization strategies (Discussion)
8. Added comprehensive security and privacy considerations (Discussion)
9. Added three new references including cardiovascular AI and advanced graph techniques

## Technical Corrections:

10. Fixed inconsistent notation (Equations 5 and 16: ℒ_struct vs. ℒ_semantic)
11. Expanded Figure 2 (Figure 3 in the revised manuscript) description to define all symbols
12. Removed journal-specific statements from Abstract
13. Streamlined Abstract and Conclusion for clarity

## Enhanced Clarity:

14. Strengthened logical connections between
15. Improved correspondence between figures
16. Added explicit definitions for all mathematical

# CONCLUSION

We believe these revisions have substantially

 **Clarity:** Improved pedagogical flow and reduced sections

**Completeness:** Added missing algorithmic analysis

**Rigor:** Fixed notational inconsistencies and

**Impact:** Better articulation of broader significance applications

 **Reproducibility:** Complete pseudocode and

We are confident that the revised manuscript now the journal and addresses all

reviewer concerns comprehensively. We once againtheir invaluable feedback that has significantly improved the quality of our work.

Sincerely,

## Masoud Amiri and Oliya Zare

Department of Biomedical Engineering Kermanshah University of Medical Sciences Kermanshah, Iran

Email: [masd.amiri@yahoo.com](mailto:masd.amiri@yahoo.com)
